# Frailty assessment and acute frailty service provision in the UK: results of a national ‘day of care’ survey

**DOI:** 10.1186/s12877-021-02679-9

**Published:** 2022-01-03

**Authors:** Thomas Knight, Catherine Atkin, Finbarr C Martin, Chris Subbe, Mark Holland, Tim Cooksley, Daniel Lasserson

**Affiliations:** 1 Department of Acute Medicine, Sandwell and West Birmingham Hospitals NHS Trust, City Hospital, B18 7QH, Birmingham, UK; 2 Birmingham Acute Care Research Group, Institute of Inflammation and Ageing, University Hospital Birmingham NHS Foundation Trust, University of Birmingham, Edgbaston, Birmingham, B15 2GW UK; 3 Population health Sciences, Kings College London, London, UK; 4 School of Medical Sciences, Bangor University & Consultant Acute, Respiratory & Critical Care Medicine, Ysbyty Gwynedd, Bangor, LL57 2PW UK; 5 Clinical and Biomedical Sciences, Faculty of Health and Wellbeing, University of Bolton, Bolton, BL3 5AB UK; 6 Manchester University Foundation Trust Oxford Rd, Manchester, M13 9WL UK; 7 Division of Health Sciences, Warwick Medical School, University of Warwick, Coventry, CV4 7AL UK

**Keywords:** Acute care, Frailty, Same day emergency care, Acute frailty unit

## Abstract

**Background:**

The incorporation of acute frailty services into the acute care pathway is increasingly common. The prevalence and impact of acute frailty services in the UK are currently unclear.

**Methods:**

The Society for Acute Medicine Benchmarking Audit (SAMBA) is a day of care survey undertaken annually within the UK. SAMBA 2019 (SAMBA19) took place on Thursday 27th June 2019. A questionnaire was used to collect hospital and patient-level data on the structure and organisation of acute care delivery. SAMBA19 sought to establish the frequency of frailty assessment tool use and describe acute frailty services nationally. Hospitals were classified based on the presence of acute frailty services and metrics of performance compared.

**Results:**

A total of 3218 patients aged ≥70 admitted to 129 hospitals were recorded in SAMBA19. The use of frailty assessment tools was reported in 80 (62.0%) hospitals. The proportion of patients assessed for the presence of frailty in individual hospitals ranged from 2.2 to 100%. Bedded Acute Frailty Units were reported in 65 (50.3%) hospitals. There was significant variation in admission rates between hospitals. This was not explained by the presence of a frailty screening policy or presence of a dedicated frailty unit.

**Conclusion:**

Two fifths of participating UK hospitals did not have a routine frailty screening policy: where this existed, rates of assessment for frailty were variable and most at-risk patients were not assessed. Responses to positive results were poorly defined. The provision of acute frailty services is variable throughout the UK. Improvement is needed for the aspirations of national policy to be fully realised.

## Background

The acute care system assesses and treats an increasing number of older patients with complex health needs. In the UK, the rate of emergency admissions for people aged 90 years and older increased by 50% between 2001/02 and 2012/13 [1]. The proportion of patients with five or more health conditions requiring emergency medical admission has also risen sharply, representing over a third of patients, compared with only one in ten a decade ago [2]. The question of how to best address the consequences of population ageing on the volume and complexity of emergency admissions is faced by many health-care systems [3].

The concept of frailty has become central to the understanding of acute illness in older people [4]. Frailty remains an evolving concept in terms of underlying biological mechanisms; however, a variety of tools can be used operationally to identify a patient cohort with poorer clinical outcomes in the acute care setting [5–8]. Frailty is associated with an increased likelihood of developing hospital acquired complications such as deconditioning and geriatric syndromes including delirium, falls, pressure ulcers and incontinence [9–13].

The NHS Long Term Plan states that all hospitals with a 24 h Emergency Department (ED) will provide an acute frailty service for at least 70 h a week, with the aim to complete a clinical frailty assessment within 30 min of a patients arrival in the ED or SDEC (Same Day Emergency Care) unit [14]. The policy is designed to reduce unnecessary hospital admissions and reduce the length of stay by providing interventions, such as comprehensive geriatric assessment (CGA) at earlier time-points within the acute care pathway.

NHS England and NHS improvement (NHSE/I) have published additional guidance detailing how the policy should be implemented and recommended metrics to measure performance. The guidance states all patients over the age of 65 should be assessed for the presence of features of frailty using the Clinical Frailty Scale (CFS), a validated tool designed to identify and grade frailty based on the severity of functional impairment [15]. Acute frailty services should be provided with the capability to deliver CGA by a multidisciplinary team containing doctors with geriatric expertise, physiotherapists, occupational therapists and case managers. Similar ambitions to improve the care for older patients living with frailty have been expressed within policy documents published by the devolved UK governments responsible for health and social care in Scotland, Wales and Northern Ireland [16–18]. The mandate to provide acute frailty services and routinely screen for frailty using the CFS is unique to England.

The means by which individual hospitals structure care to achieve these policy objectives is at the discretion of the organisation. There are two broad care models by which acute frailty services can be delivered. Assessment can be provided within designated Acute Frailty Units (AFU) which provide bed-based care, or mobile “in-reach” teams which typically provide CGA in the ED. [19–22] These models are not mutually exclusive.

We aimed to describe the extent to which frailty identification is undertaken within a cross-section of UK hospitals and describe variations in the structures and processes employed. We also aimed to assess whether the implementation of these interventions was associated with differences in the number of patients discharged without overnight stay and the number of patients admitted for longer than 7 days.

## Methods

The Society for Acute Medicine Benchmarking Audit (SAMBA) is a day-of-care survey conducted by the Society for Acute Medicine on the penultimate Thursday in June on an annual basis. The UK wide survey collects data on the organisational structure of participating hospitals and adherence to national guidelines. The survey also collects patient-level data relating to the process of care and outcomes assessed 7 days after admission. SAMBA 2019 (SAMBA19) was completed on Thursday 27th June 2019 between 00:00 and 23:59. The SAMBA19 protocol is publicly available [23]. The SAMBA19 main report is published elsewhere [24].

Recruitment to SAMBA19 was open to all hospitals in the UK receiving acutely unwell (non-elective, adult) medical patients. Non-acute and community hospitals were excluded. All patients referred for emergency or unscheduled medical care during this period were eligible for inclusion. Before patient-level data collection, a designated clinician in each participating hospital completed an online questionnaire containing questions relating to the presence and structure of acute frailty services. The questionnaire established whether a frailty assessment tool was in routine use, the specific tool used, and what criteria triggered assessment at an organisational level.

Patient level analysis was restricted to patients aged ≥70. Whether the patient had been screened for frailty and the outcome of assessment was recorded. The outcome of frailty assessment was recorded as a dichotomous variable (frail vs non-frail) defined by the local thresholds in operation at the hospital. Not all hospitals routinely employed a frailty assessment tool, and not all patients within hospitals that reported use of a tool were assessed for the presence of frailty. To maximise the use of available data when the outcome of frailty assessment was not recorded, receipt of any form of formal care package or admission from care home (residential or nursing) was used as a proxy for frailty. Patient outcomes were recorded by review of the case notes or electronic health record at 7 days after admission.

The proportion of patients discharged on the same day as admission is reported from a sub-group of patients with a National Early Warning Score 2 (NEWS2) less than five as crude adjustment for severity of acute illness. A NEWS2 < 5 has been proposed as a consensus threshold below which same-day discharge may be considered appropriate in selected patients [25, 26].

## Statistical analysis

Analysis was restricted to hospitals that submitted both organisational and patient-level data. Missing values were not imputed. Continuous data is described using the mean and standard deviation for normally distributed data and the median and interquartile range (IQR) for non-normally distributed data. Outcome variables were described using counts and proportions with 95% confidence intervals (CI). Hospitals were grouped based on acute frailty services and box and whisker plots used to demonstrate the variation in the outcome measure at the hospital level. Analysis was performed using R statistical software (Version 1.3.1093, Vienna. Austria).

## Results

A total of 139 UK hospitals contributed data to SAMBA19; 10 hospitals contributed patient data without paired organisational data and were excluded. Analysis was therefore undertaken on data obtained from 129 individual hospitals. Patient level data was available from 3218 individuals aged ≥70, this represented 47.6% of the total number of patients recorded in SAMBA19 (*n* = 6756). The majority of patients, 2849 (88.5%) were admitted directly from their own home, 310 (9.6%) were admitted from a nursing or residential care setting and 600 (18.6%) patients were in receipt of a package of care in their own home. The relative proportion of patients aged ≥70 of the total number of patients within each hospital ranged from 10.7 to 76.9%. A summary of hospital characteristic by country is provided in Table 1.

**Table 1:** Summary of participating hospitals stratified by country

Characteristic	Overall, *N* = 129^a^	England, *N* = 114^a^	Wales, *N* = 6^a^	Scotland, *N* = 5^a^	Northern Ireland, *N* = 4^a^
**Inpatient hospital beds**	519 (376,709)	537 (365,706)	470 (432, 552)	725 (452, 900)	299 (173,424)
**Number of patients admitted per hospital**	49 (34,67)	52 (35,68)	36 (31, 39)	49 (29,73)	24 (12,35)
**Number of patients over 70 per hospital**	24 (16, 31)	24 (17,34)	16 (14, 17)	27 (16, 13)	16 (9,22)
**Frailty assessment tool use**	80 (62%)	73 (64%)	1 (17%)	5 (100%)	1 (25%)
**Bedded AFU**	63 (49%)	60 (53%)	0 (0%)	2 (40%)	1 (25%)
**ED in reach**	62 (48%)	57 (50%)	1 (17%)	3 (60%)	1 (25%)
**Frailty service active > 70 h a week**	29 (22%)	26 (23%)	0 (0%)	3 (60%)	0 (0%)

^a^Median (IQR);n (%)

### Frailty screening

A policy to perform frailty screening was reported by 80 (62.0%) hospitals. The use of frailty assessment tools was dependent on the route of entry into the acute care pathway.

Frailty assessment was undertaken in the ED in 50 (62.5%) hospitals and on the Acute Medical Unit (AMU), a dedicated short stay ward for receiving medical admissions, in 48 (60.0%) hospitals. The use of frailty assessment tools in designated ambulatory emergency care areas (a specific location within the hospital designed to deliver SDEC) was recorded by 14 (17.5%) hospitals.

CFS was used most frequently as an assessment tool, accounting for 62 (77.5%) of responses, 4 (5.0%) hospitals used the abbreviated Comprehensive Geriatric Assessment, 4 (5.0%) hospitals used the Health Improvement Scotland “Think Frailty” tool [27] and 1 (1.3%) hospital used the Edmonton Frail Scale [28]. Locally developed screening tools were used in 4 (5.0%) hospitals. Locally developed tools included the modified Bournemouth criteria in 3 hospitals and the Leicester criteria in 1 hospital. Details of these locally developed tools were not recorded in the survey or available from the published literature, The specific tool used was not recorded by 5 (6.3%) hospitals.

The patient characteristics which triggered frailty assessment varied between hospitals. Age was the most common trigger, used in 47 (58.8%) hospitals, followed by presenting syndrome in 17 (21.3%) hospitals. Specific details of the age threshold or presenting syndrome used to trigger assessment were not recorded. The population targeted for frailty assessment was not specified in 16 (20.0%) hospitals. The actions taken in response to the identification of frailty was not formalised within a policy document in 38 (47.5%) of the hospitals that reported the use of a frailty assessment tool.

**Fig. 1:**
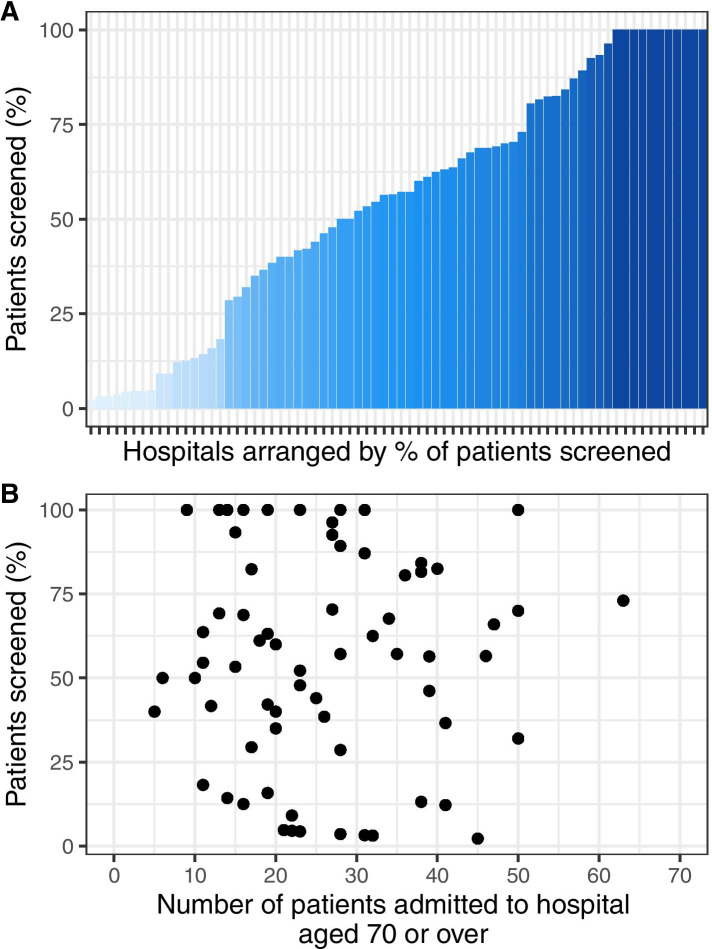
**A** Bar plot summarising the proportion of patients aged ≥70 screened within participating hospitals **B** Scatter plot demonstrating the relationship between the number of patients aged ≥70 admitted to each individual hospital and the proportion of patients assessed for frailty

In total, 2051 patients were admitted to hospitals that reported the use of a frailty assessment tool. Frailty assessment was performed in 1026 (50.0%). There was significant variation in the proportion of patients assessed at the hospital level, ranging from 2.2 to 100%. (Fig. 1A) There was no correlation between the number of patients aged ≥70 and the proportion of patients assessed for frailty within each hospital. (Fig. 1B).

**Fig. 2:**
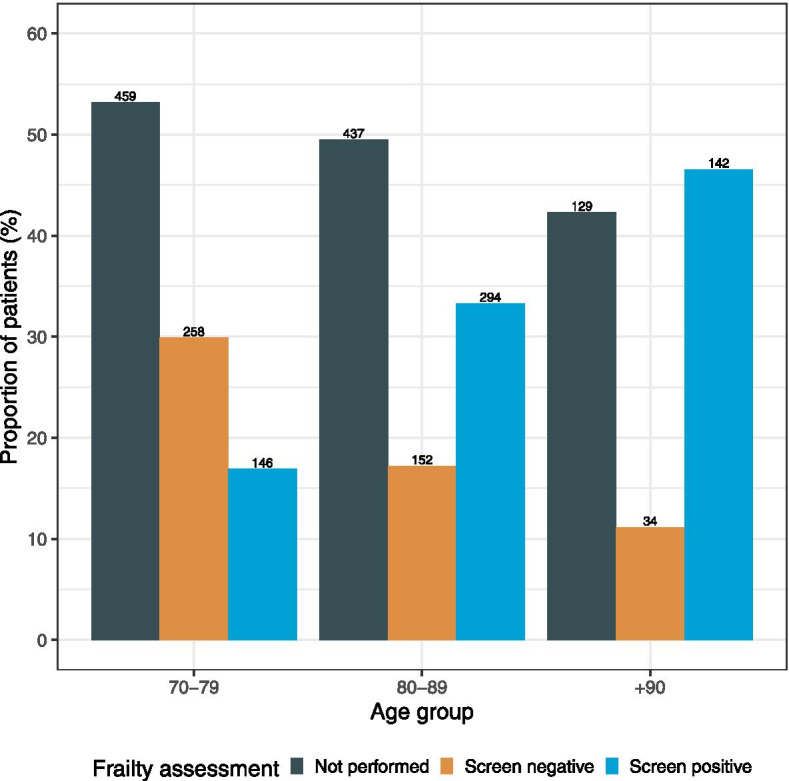
Bar plot summarising number of patients screened for frailty and the outcome of screening stratified by age

Frailty was identified in 582 (56.6%) patients. The proportion of patients assessed, and the outcome of assessment stratified by age in hospitals that reported the use of a frailty assessment tool is provided in Fig. 2. Frailty assessment was undertaken in 404 (44.6%) patients in the 70–79 age group compared with 446 (50.5%) in the 80–89 age group (*p* value = 0.01).

### Acute frailty services

**Fig. 3:**
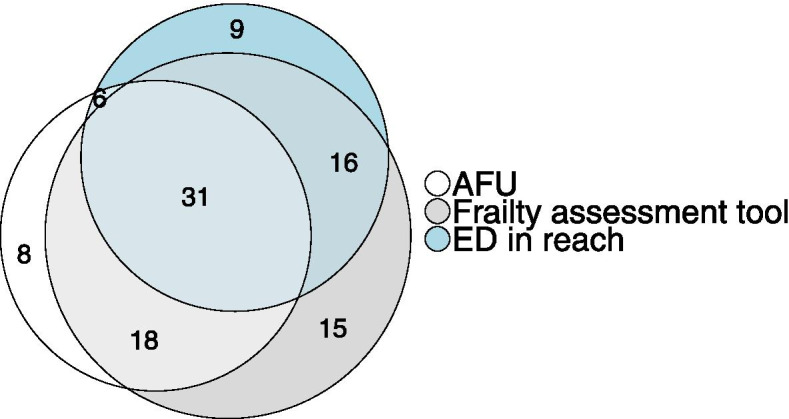
Venn diagram demonstrating the provision of acute frailty services and use of frailty assessment tools

Provision of an AFU was reported in 63 (48.9%) hospitals: 18 (28.6%) were located within the AMU and 45 (71.4%) were geographically separated. Acute frailty services were provided using an in-reach model in 62 (48.1%) hospitals. In-reach, services were provided in addition to a specific bedded AFU in 37 (28.7%) hospitals. Acute frailty services were operational for more than 70 h in 29 (14.6%) hospitals. A Geriatrician led the AFU in 57 (87.7%) hospitals. The combination of different acute frailty services and association with the use of frailty assessment tools at the hospital level is shown in Fig. 3.

### Outcomes

**Fig. 4:**
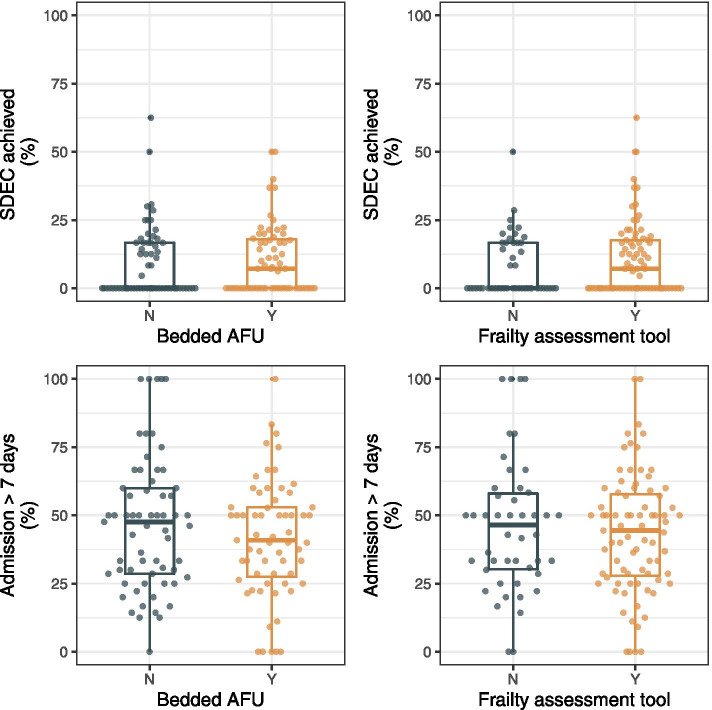
Boxplots demonstrating the proportion of patients achieving same day discharge and the proportion of patients requiring inpatient admission > 7 days within individual hospitals stratified by use of frailty assessment tools and the presence of AFUs

The impact of different acute frailty services on outcome was assessed in 1179 (36.6%) patients with features suggestive of frailty (positive frailty assessment, *n* = 582; admission from care home, *n* = 166; or prior receipt of a formal package of care, *n* = 431). The median number of patients per hospital was 8 (IQR 3–13). In-patient admission of longer than 7 days occurred in 510 (43.3, 95%CI 40.5–46.1) cases. There was significant variation in the proportion of patients admitted for longer than 7 days at the hospital level. The variation did not appear to be explained by the use of frailty assessment tools or the presence of an AFU (Fig. 4).

The NEWS2 was < 5 in 958 (81.3%) patients with features suggestive of frailty. In this group, same-day discharge was achieved in 121 (12.6, 95%CI 10.7–14.9) patients. The proportion of patients with frailty discharged on the same day ranged from 0.0 to 62.0% within individual hospitals. No discharges on the same day of admission were observed in 63 (48.8%) hospitals. Variation did not appear to be explained by the use of frailty assessment tools or the presence of AFUs (Fig. 4).

## Discussion

We report the results of a nationwide survey that provides insight into the current design and implementation of adaptations to the acute care pathway for older patients living with frailty. We observed a significant gap between the current provision and the aspirations of national policy. The majority of hospitals have incorporated a frailty assessment tool into the acute care pathway, but many have not defined the steps to take when frailty is identified.

The provision of acute frailty services is not uniform across the UK. The responsibility for healthcare delivery by the NHS is devolved to the individual governments of the UK, this complicates the policy landscape. Acute frailty services were less common in Northern Ireland and Wales, possibility reflecting the absence of clear policy mandate in these countries. The prevalence of acute frailty services was highest in England and Scotland. This may be explained by the activity of improvement programs such as the acute Frailty Network and the Frailty at the Front Door Collaborative operating within these countries [29, 30]. These initiatives provide interventions designed to enable local hospitals to adopt new frailty services and processes by providing support from national clinical and improvement experts as well as a forum to share resources and best practice.

The explicit commitment to acute frailty services and frailty assessment in the NHS Long term plan is likely to have driven adoption in England. Despite this, many hospitals in England have not yet complied with the policy mandate. This may represent the time lag between policy and the commissioning of new services. Workforce shortages within the NHS are well documented and the skills required to deliver acute frailty services are a relatively scarce resource. The implementation strategy has, so far, focused on engaging clinical leaders, communicating with stakeholders and disseminating good practice. This approach balances central policy against local autonomy. Identifying barriers to implementation in hospitals that do not currently provide these services may help drive system wide change. The pressure to change at the local level will mount if the absence of appropriate services is associated with measurable differences in performance and outcomes.

In hospitals where a frailty assessment tool was stated policy, the use of the tool was inconsistent. We observed wide variation in the proportion of patients assessed using the tool between hospitals. The disparity between the number of patients assessed for frailty and the number of patients at risk has previously been described [31]. Frailty assessment is feasible in acutely unwell patients, [32] and surrogates can provide equivalent information when the patient cannot participate in the process directly [33].. Mandating frailty assessment at the policy level and adopting the policy at the hospital level does not guarantee translation at the patient level. Embedding frailty assessment into existing clinical pathways requires a degree of cultural change and recognition of the importance of identifying frailty by those delivering care. The extent to which potential deficiencies in the tools themselves might have contributed to the observed variation remains to be further investigated.

Frailty assessment tools are not inherently beneficial. Identifying frailty is only of benefit if it results in a differential response that positively impacts patient care. Increasing the prevalence of frailty assessment should not be seen as an end in itself. Frailty assessment tools should be judged based on their ability to alter downstream care and trigger targeted interventions. Frailty assessment tools have been evaluated extensively for their ability to predict adverse outcomes, but, how best to operationalise the information obtained is less well established [34]. The use of frailty assessment tools can prime the acute care pathway with information that allows subsequent care to be better calibrated to the patient’s needs. Accurate risk stratification at the point of triage allows early intervention in patients most at risk of deterioration and the opportunity to alter the trajectory of illness while simultaneously expediting the care of patients at the lower end of the risk spectrum. Incorporating frailty assessment has previously been shown to yield efficiencies in the average length of stay among patients at low clinical deterioration risk [35].

The emergence of coronavirus disease 2019 has radically altered the delivery of acute care. The National Institute of Clinical Excellence has advocated using CFS as a tool to aid decision making concerning treatment escalation and the provision of advanced respiratory support [36]. It will be interesting to determine through future iterations of SAMBA whether advocacy for the use of the CFS during the pandemic has led to a sustained increase in usage in routine clinical practice.

The proportion of older patients with frailty discharged on the same day as admission varied widely between hospitals. A significant proportion of hospitals did not discharge a single patient on the same day as admission. The cause of this variation is unclear. Aggregating hospitals based on specific services did not appear to explain the observed variation, but this simple approach lacks the sensitivity required to detect meaningful differences in outcome. Acute frailty services represent one element of a system-wide response to frailty, intrinsically linked to social care and community services. The success of acute frailty services is likely to be moderated by how well these elements interact. The hospital-level data within SAMBA19 lack the granularity to explore these factors in further detail.

### Limitations

In addition to the limitations discussed, several other factors should be considered when interpreting our study. Hospital and patient-level data were self-reported, which may influence objectivity. Hospitals are provided with a detailed summary of their individual performance, but hospitals are anonymized for all other purposes, potentially reducing the risk of biased reporting. There were 225 hospitals eligible to participate in SAMBA19 [37]. The response rate of 57.3% allows a reasonable but not complete evaluation of the variation in the organisational approach to frailty at the system level. There may be systemic differences between participating and non-participating hospitals.

We did not employ extensive measures of case-mix adjustment, which could account for differences in outcomes. We attempted to take account of the severity of physiological disturbances when assessing the proportion of patients discharged on the same day as admission. This approach does not take into account other influential covariates. The population classified as frail within our analysis is likely to be heterogenous given the varying criteria used to trigger assessment and the different tools employed at each hospital.

Defining frailty as a dichotomous outcome variable based on local criteria precluded analysis based on the severity of frailty. Threshold values used to define frailty may have differed significantly at the hospital level. We were unable to identify whether individual patients had direct interaction with acute frailty services. It is possible that AFUs had a significant impact at the patient level that could not be measured when combined with patients managed independently of the service.

## Conclusion

Older patients living with frailty frequently require acute medical care during times of crisis. We highlight significant differences in the provision of acute frailty services across the UK. Many hospitals do not currently use a frailty assessment tool or provide acute frailty services. In trusts with systems in place to identify frailty, many at-risk patients are not screened, and responses, when frailty is identified, are currently poorly defined. At the system level, frailty assessment tools and AFU were not associated with differences in the proportion of patients discharged on the same day as admission or admitted for > 7 days.

## Data Availability

The data that support the findings of this study are available from the Society for Acute Medicine, but restrictions apply to the availability of these data, which were used under license for the current study, and so are not publicly available. Data are however available from the authors upon reasonable request and with permission of the Society for Acute Medicine.

## Abbreviations

AFU: Acute Frailty Unit
AMU: Acute Medical Unit
CI: Confidence interval
CFS: Clinical Frailty Scale
CGA: Comprehensive geriatric assessment
ED: Emergency Department
IQR: Interquartile range
NEWS2: National Early Warning Score 2
NHSE/I: NHS England NHS Improvement
SAMBA: The Society for Acute Medicine Benchmarking Audit
SDEC: Same Day Emergency Care
